# *In vitro* Assays and Imaging Methods for Drug Discovery for Cardiac Fibrosis

**DOI:** 10.3389/fphys.2021.697270

**Published:** 2021-07-08

**Authors:** Giorgia Palano, Ariana Foinquinos, Erik Müllers

**Affiliations:** ^1^Division of Physiological Chemistry I, Department of Medical Biochemistry and Biophysics, Karolinska Institutet, Stockholm, Sweden; ^2^Bioscience Cardiovascular, Research and Early Development, Cardiovascular, Renal and Metabolism, BioPharmaceuticals R&D, AstraZeneca, Gothenburg, Sweden

**Keywords:** cardiac fibrosis, *in vitro* assays, 3D models, co-culture systems, high-content imaging, drug discovery

## Abstract

As a result of stress, injury, or aging, cardiac fibrosis is characterized by excessive deposition of extracellular matrix (ECM) components resulting in pathological remodeling, tissue stiffening, ventricular dilatation, and cardiac dysfunction that contribute to heart failure (HF) and eventually death. Currently, there are no effective therapies specifically targeting cardiac fibrosis, partially due to limited understanding of the pathological mechanisms and the lack of predictive *in vitro* models for high-throughput screening of antifibrotic compounds. The use of more relevant cell models, three-dimensional (3D) models, and coculture systems, together with high-content imaging (HCI) and machine learning (ML)-based image analysis, is expected to improve predictivity and throughput of *in vitro* models for cardiac fibrosis. In this review, we present an overview of available *in vitro* assays for cardiac fibrosis. We highlight the potential of more physiological 3D cardiac organoids and coculture systems and discuss HCI and automated artificial intelligence (AI)-based image analysis as key methods able to capture the complexity of cardiac fibrosis *in vitro*. As 3D and coculture models will soon be sufficiently mature for application in large-scale preclinical drug discovery, we expect the combination of more relevant models and high-content analysis to greatly increase translation from *in vitro* to *in vivo* models and facilitate the discovery of novel targets and drugs against cardiac fibrosis.

## Introduction

Cardiac fibrosis is a common feature in the pathology of many forms of cardiovascular diseases. It is characterized by excessive production and deposition of extracellular matrix (ECM) components in the connective tissue of the heart, mainly collagens, that confer stiffness and loss of contractility, thereby reducing cardiac function (reviewed in Kong et al., 2014; Liu et al., 2017).

At the cellular level, the main cells responsible for cardiac fibrosis are cardiac fibroblasts (CFs), which are producing collagen and other ECM proteins. Following cardiac injury, CFs become activated and can differentiate into myofibroblasts (myoFBs), a cell type exhibiting stress fibers and characterized by the expression of alpha-smooth muscle actin (α-SMA) and other contractile proteins (Gabbiani et al., 1971; Skalli et al., 1989). It is well known that one of the molecular mechanisms driving cardiac fibrosis is the transforming growth factor-beta (TGF-β) signaling pathway. TGF-β is a potent inducer of collagen synthesis that can induce phenotypic changes in CFs and their differentiation into myoFBs (Desmouliere et al., 1993). MyoFBs secrete high levels of ECM proteins, such as collagens, including collagen type I, which is the most abundant protein of the cardiac ECM and the main component of fibrotic tissue. It is synthesized as a collagen precursor (procollagen) that is processed to form mature collagen, which is released into the extracellular space (reviewed in Canty and Kadler, 2005). The excessive accumulation of mature collagen and deposition of ECM can lead to either reparative fibrosis or reactive fibrosis. Reparative fibrosis is mainly seen after an acute myocardial infarction (MI) to replace dead cardiomyocytes with fibrotic scar tissue or in end-stage heart failure (HF). Also, reactive fibrosis is seen after MI and HF, but it mainly occurs as a pathological condition related to age and HF with preserved ejection fraction (HFpEF) (Heymans et al., 2015).

Preclinical studies indicate that cardiac fibrosis can be attenuated, reduced, and even reversed by the pharmaceutical intervention (Nagaraju et al., 2019). Thus, there is an enormous interest to find new treatments for cardiac fibrosis and to better understand its pathogenesis. New promising pathways involving several noncoding RNAs, such as microRNA 21 (miRNA-21), miR-208a, nuclear enriched abundant transcript 1 (Neat1), and maternally expressed 3 (Meg 3), have emerged in preclinical studies. Also, candidates like galectin-3 (Gal-3), neutrophil gelatinase-associated lipocalin (NGAL), cardiotrophin-1 (CT-1), and NADPH oxidase 2 (NOX2) have been associated clinically and preclinically with collagen dysregulation (Sharma et al., 2004; Yndestad et al., 2009; Murdoch et al., 2014; Heymans et al., 2015; Piccoli et al., 2017; Kenneweg et al., 2019).

Physiologically relevant, predictive, and reliable *in vitro* assays are required to study the underlying molecular mechanisms and to find drugs against cardiac fibrosis. In this study, we review different assays and imaging methods to detect cardiac fibrosis *in vitro*, and we discuss the challenges and future directions of the development of novel assays.

## *In vitro* Assays for Cardiac Fibrosis

### Challenges Assessing Cardiac Fibrosis *in vitro*

Cardiac fibrosis is a complex, multistep cellular and molecular process that takes weeks to months *in vivo* and has, thus, proven difficult to recapitulate *in vitro*. Progress to develop physiologically relevant cardiac cell culture models has been slower compared to other organ tissues, for example, kidney, liver, or tumors. The discovery of treatments for cardiac fibrosis has, therefore, been hampered by the lack of reliable and predictive *in vitro* assays with sufficient throughput for drug discovery. An ideal cardiac fibrosis assay needs to recapitulate multiple, highly complex processes, such as:

(a) Formation of myoFBs, which differentiate from fibroblasts or epithelial to mesenchymal trans-differentiation upon induction of cytokine, and differentiation to CFs (Bryant et al., 2009; Li et al., 2016).
(b) Activation/differentiation of fibroblasts, driven by multiple cytokine stimuli such as TGF-β, angiotensin II, platelet-derived growth factor (PDGF), endothelin-1, tumor necrosis factor-alpha (TNFα), interleukin-1 beta (IL-1β), and others, inflammation-mediated or triggered by mechanical stress (reviewed in Aujla and Kassiri, 2021).
(c) Activation of multiple canonical (e.g., Smad3/4) and noncanonical [e.g., transforming growth factor-β-activated kinase 1 (TAK1), c-Jun N-terminal kinase (JNK), phosphoinositide 3-kinase (PI3K), and Ras homolog family member A (RhoA) (reviewed in Zhang, 2017) signaling pathways, resulting in the induction of fibrogenic gene transcription] encoding proteins such as α-SMA, collagen, matrix metalloproteinases (MMPs), and connective tissue growth factor (CCN2), which increase deposition of collagen and other ECM proteins.
(d) Production, secretion, processing, and maturation of ECM proteins such as collagens (Canty and Kadler, 2005).

**FIGURE 1:**
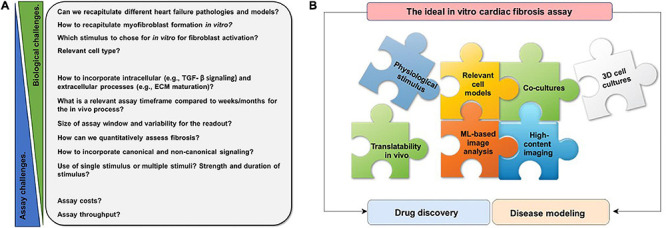
Schematic overview of challenges of recapitulating cardiac fibrosis *in vitro* and features of an ideal *in vitro* cardiac fibrosis assay. **(A)** Numerous challenges have to be overcome to recapitulate the complex, multistep process of cardiac fibrosis *in vitro*. These are challenges of fibroblast biology, challenges in assay development, or in between. **(B)** Each puzzle piece represents a feature that should be taken into account for the ideal *in vitro* cardiac fibrosis assay. Many different aspects, such as physiological stimulus, relevant cell models, cocultures, three-dimensional (3D) cell culture, high-content imaging, machine learning (ML)-based analysis, translatability to *in vivo*, and need to be considered for a physiologically relevant *in vitro* cardiac fibrosis assay.

Thus, there are multiple challenges to recapitulating cardiac fibrosis in a dish (Figure 1A). The ideal cardiac fibrosis assay should build on physiologically relevant cells and needs to incorporate relevant readouts. In order to be applicable for drug screening, it needs to be robust, cost- and time-effective, and high-throughput. If possible, it should recapitulate the effects of three-dimensional (3D) structure and incorporate multiple relevant cell types involved. Finding the ideal *in vitro* assay is a complex puzzle of many pieces (Figure 1B).

### Readouts of Cardiac Fibrosis Assays

**FIGURE 2:**
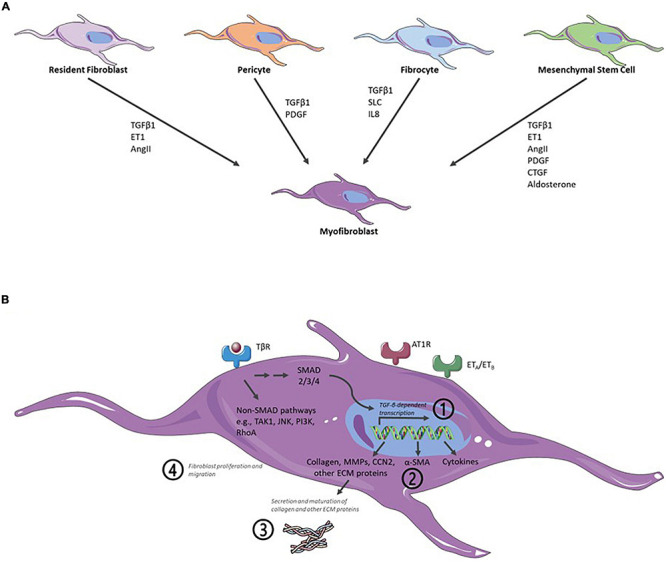
Schematic representation of major biochemical pathways involved in the formation of myofibroblasts (myoFBs), activation of cardiac fibroblasts (CFs), profibrotic signaling, and key readouts for *in vitro* assays. **(A)** *In vivo* formation of myoFBs occurs from different cell types including resident fibroblasts, pericytes, fibrocytes, or mesenchymal stem cells, stimulated by mediators such as TGFβ1, ET1 (endothelin 1), AngII (angiotensin II), PDGF (platelet-derived growth factor), SLC (secondary lymphoid chemokine), IL8 (interleukin-8), CTGF (connective tissue factor), or aldosterone. **(B)** Activation of CFs is triggered by a myriad of cytokines and growth factors, such as TGF-β, CCN2 (connective tissue factor 2), PDGFs, AngII, aldosterone, endothelin-1, TNFα (tumor necrosis factor α), IL1-β (interleukin-1 β), or IL6 (interleukin-6). Activation of TGF-β receptor (TβR) leads to activation of downstream canonical SMAD and noncanonical pathways. The majority of cardiac fibrosis assays rely on measuring one of four major readouts: (Kong et al., 2014) TGF-β pathway activation/TGF-β-dependent gene expression; (Liu et al., 2017) α-SMA expression; (Gabbiani et al., 1971) (mature) collagen detection; and (Skalli et al., 1989) fibroblast proliferation or migration. Angiotensin II receptor type 1-mediated (AT1R) signaling and endothelin receptor-mediated (ET_*A*_/ET_*B*_) signaling also contribute to cardiac fibrosis. As an excellent, in-depth review of signaling for the formation of myoFB and activation of CF, the reader is referred to the study of Aujla and Kassiri (2021). Figure created using Servier Medical Art by Servier (https://smart.servier.com/), licensed under a Creative Commons Attribution 3.0 Unported License.

Naturally, *in vitro* assays can only recapitulate parts of the *in vivo* situation. MyoFB is central to cardiac fibrosis and, thus, to any cardiac fibrosis *in vitro* assay. Formation of myoFB and activation of fibroblast can result from different cell types and integrating several major biochemical pathways (Figure 2A). In principle, any of these pathways and their downstream signaling can provide the basis for an assay readout. Notably, an *in vitro* assay is often limited to a single or few cell types (see sections “Cell Models” and “Three-Dimensional Models and Coculture Systems”), therefore, limiting the possibility of paracrine fibroblast activation. In fibroblast-only cell culture, TGF-β is the major pathway for activation of fibroblast and the stimulus of choice for the majority of cardiac fibrosis assays (Figure 2B).

Current cardiac fibrosis assays can broadly be divided into four groups based on their main readout: (a) TGF-β pathway activation/TGF-β-dependent gene expression; (b) α-SMA expression; (c) (mature) collagen detection; and (d) fibroblast proliferation or migration (Figure 2B and Table 1).

**TABLE 1:** Examples of assays to assess features of cardiac fibrosis *in vitro*.

**Assay readout**	**Method (example references)**	**Advantages**	**Limitations**
TGF-β-dependent gene expression	RT-qPCR (Ranganathan et al., 2007)	• High sensitivity • Quantitative analysis	• Often measurement of a single/a few gene(s) only

TGF-β dependent gene expression	Reporter cell lines (Tesseur et al., 2006)	• High-throughput^*a*^ • Quantitative analysis	• Bias to a single promoter readout • Requires cell line engineering

α-SMA protein expression	IF staining (Shinde et al., 2017; Baranyi et al., 2019)	• Allows assessment of intracellular localization and single-cell analysis • Quantitative analysis • Can be set up in high-throughput • Sensitive to changes in cell number and density	• Requires quantitative fluorescence microscopy instruments • Dependent on good, specific immunoreagents • Requires only low cell number

Collagen protein expression	Western blot	• Detection of different forms and type • Semi-quantitative analysis	• Antibodies exist only in specific forms and types of collagen • Highly dependent on immunoreagents • Requires larger amounts of cells

Collagen protein expression	ELISA (Baranyi et al., 2019)	• High sensitivity and specificity • Quantitative analysis • Can be set up in high-throughput • Requires less material than WB	• Dependent on immunoreagents

Visualization of collagen fibers	Sirius Red dye (Junqueira et al., 1979; Whittaker et al., 1994)	• Mature collagen fiber formation is a late hallmark of cardiac fibrosis • Semi-quantitative analysis	• *In vivo* method that is difficult to adapt to *in vitro*

Visualization of collagen fibers	Masson’s trichrome staining	• Mature collagen fiber formation is a late hallmark of cardiac fibrosis • Semi-quantitative analysis	• *In vivo* method that is difficult to adapt to *in vitro*

Collagen peptide detection	PIP (Querejeta et al., 2000, 2004)	• *In vivo* biomarker that can be adapted to *in vitro* • Quantitative analysis • Potentially high-throughput	• Bias to a single-marker readout that is not fully specific for cardiac fibrosis

Direct visualization of collagen fibers	Electron microscopy	• Mature collagen fiber formation is a late hallmark of cardiac fibrosis	• No quantitative analysis • Requires electron microscopy equipment and expertise, usually only available in highly specialized laboratories • Low throughput • Does not distinguish different types and forms

Hydroxyproline quantification	HPCL/LC-MS (Qiu et al., 2014)	• *In vivo* biomarker that can be adapted to *in vitro* • Quantitative analysis	• Requires HPLC capabilities, mass spectrometry equipment, and high-level of technology-specific expertise • Bias to a single-marker readout that is not specific for cardiac fibrosis

Collagen detection	MS (van Huizen et al., 2020)	• Possible to detect different forms and types of collagen • Semi-quantitative analysis	• Low throughput • Requires mass spectrometry instrument and high-level of technology-specific expertise

Cardiac fibroblast migration	Cell migration assay (scratch assay) (Liang et al., 2007)	• Semi-quantitative analysis • Medium throughput	• Can be adapted for 3D and coculture models

Cardiac fibroblast proliferation	Cell count or proliferation markers	• Quantitative analysis • Low cost and high-throughput • Adaptable for IF staining and imaging • Can be combined with ELISA and/or flow cytometry • Can be adapted for 3D and coculture models	• Counter-screening required to identify fibroblast-specific proliferators • Fibroblast proliferation is not predictive for cardiac fibrosis

Multiparametric readout, i.e., phenotypic fingerprints	Transcriptomics or proteomics (Barallobre-Barreiro et al., 2012; Abonnenc et al., 2013; Jonsson et al., 2016)	• Multiple parameters integrated to score fibrosis phenotype • Allows for unbiased detection of also unknown phenotypes	• Necessary instrumentation is usually only available in highly specialized laboratories • Requires infrastructure for data handling and specific expertise for data analysis • High cost and low throughput • Little precedence for *in vitro* application • Readouts can be difficult to interpret

Multiparametric readout, i.e., phenotypic fingerprints	High-content imaging (Pantazis et al., 2010; Palano et al., 2020)	• Multiple parameters integrated to score fibrosis phenotype • Allows for unbiased detection of also unknown phenotypes • Low cost and high-throughput • Single-cell analysis	• Requires quantitative fluorescence microscopy instruments • Requires infrastructure for data handling and specific expertise for data analysis

*^*a*^An assay is considered high-throughput if it can generally be run in 96-well format at reagent costs > 1$ per data point.*

Due to the complexity of fibrosis *in vivo*, it is very difficult to mimic with a single *in vitro* assay. Typically, each assay aims to model a key event in the fibrotic pathway, such as myoFB differentiation, TGF-β pathway activation, or collagen production. These assays are well suited to capture the respective parts of the fibrotic process and to evaluate specific effects of compounds *in vitro;* nonetheless, the current assays have a poor history of finding hits that translate to clinical applications. The reasons for this are manifold. Target- or biomarker-specific assays capture a single process very well but do not capture cardiac fibrosis as a whole. Reporter cell lines for TGF-β-dependent gene expression allow for robust, cell-based, and high-throughput screening (Tesseur et al., 2006). However, the engineered cells do not fully reflect the physiology of CFs. Hits are likely limited to the TGF-βreceptor → SMAD3/4 → TGF-β-dependent gene expression signaling axis and, thus, limited to early signaling nodes and TGF-β as a stimulus. Such assays can provide valuable information for target deconvolution, i.e., they can indicate or exclude specific target/pathway activity if the target or mechanism of action for a hit compound is not known. As such, they should be integral parts of a drug-screening cascade for cardiac fibrosis.

Similarly, assays that measure biomarkers, such as quantification of PICP (propeptide of procollagen type I) or hydroxyproline, can provide valuable information in a drug-screening cascade. Assessing these biomarkers *in vitro* increases the understanding of the mechanism of action of a compound and, thus, improves the chances for *in vivo* translation. As such, these types of assays are best suited for not only initial high-throughput screening (HTS) but also downstream profiling of hits.

Alpha-smooth muscle actin protein expression is an early hallmark in cardiac fibrosis. Many high-quality immunoreagents are available, and thus, especially immunofluorescence of α-SMA has emerged as an attractive readout to screen for compounds with antifibrotic activity in the heart (Rehman et al., 2019) or other tissues (Sieber et al., 2018; Weigle et al., 2019). Formation and secretion of mature collagen follow further downstream of TGF-β and α-SMA (Figure 2B). Quantification of mature collagen integrates upstream signaling, and, as a major component of excessive production of ECM, collagen contributes to the *in vivo* pathomechanism. However, there are many different forms of collagen and assays and reagents to specifically and quantitatively assess whether mature collagen is sparse. Interestingly, several recent screening approaches utilize immunofluorescence of both α-SMA and collagen, which could be seen as the first step to a multiparametric approach (Weigle et al., 2019; Turner et al., 2020).

Schimmel et al. (2020) analyzed three readouts, namely, the proliferation of human CFs, modulation of apoptosis, and expression of ECM, when screening a natural compound library for molecules for the treatment of cardiac fibrosis. Sieber et al. (2018) combined high-content imaging (HCI) with an impedance-based assay and multiplexed quantification of α-SMA and collagen 1α. The incorporation of multiple readouts adds additional information and reduces the intrinsic bias in any screening assay (Abraham et al., 2014). Omics-type approaches, such as transcriptomics, proteomics, and phenomics (i.e., HCI), are phenotypic drug discovery approaches and as such provide the opportunity to discover novel targets or compounds with novel mechanisms of action. Although less biased and very rich in information, they require special infrastructure, expertise, and resources to deconvolute the hits from a screen (also see section “High-Content Imaging”).

### Cell Models

The *in vitro* cell model is of high importance for antifibrotic effects to translate to *in vivo*. More physiologically relevant cell models often come with higher complexity and, thus, lower throughput for drug screening (Horvath et al., 2016; Table 2). The NIH 3T3 murine fibroblast cell line is a widely used cell line for proof-of-concept studies due to their high cell proliferation rate and easy handling in cell culture. TGF-β1 treatment of NIH 3T3 fibroblasts results in transformation into activated myoFBs with characteristics of α-SMA (Desmouliere et al., 1993) and collagen expression (Grande et al., 1997). Primary fibroblasts isolated from rat or murine hearts are an alternative model that many researchers chose for their translational advantages (Sahadevan and Allen, 2021). Using primary cells requires specific techniques of cell isolation and culture to maintain them for a limited amount of passages. Special care is to be taken as cultured primary fibroblasts differentiate into myoFBs *in vitro* upon prolonged culture and repeated passaging (Santiago et al., 2010). From a translational point of view, human primary fibroblasts derived from cardiac tissue are the leading choice. They are commercially available and allow us to study the remodeling of both physiological and pathological cardiac matrix. However, primary fibroblasts can be transformed after prolonged culture (Baranyi et al., 2019). Other important factors to consider when using primary fibroblasts in drug screening campaigns are the need for supply of large amounts of cells and donor-to-donor variations, i.e., the risk that findings might be specific to a certain donor. The use of immortalized primary CFs can mitigate some of these issues and thus reduce assay variability.

**TABLE 2:** Cell models used in *in vitro* cardiac fibrosis assays.

**Cell model**	**Advantages**	**Limitations**
NIH 3T3 murine fibroblasts	• High cell proliferation rate • Easy handling • Unlimited cell supply	• Limited translation to *in vivo* studies

Primary rat or murine cardiac fibroblasts	• Higher physiological relevance • Better translation to *in vivo* as mouse or rat is often the first *in vivo* model	• Requires specific techniques of isolation and culture • Cells differentiate to myofibroblasts upon prolonged culture • Limited amount of passages

Primary human cardiac fibroblasts	• Higher likelihood for translation to clinical studies • Allow to study physiological and pathological matrix remodeling	• Can be transformed after prolonged culture • Limited cell supply • High costs • Donor-to-donor variations

Immortalized human cardiac fibroblasts	• Higher likelihood for translation to clinical studies • Larger amount of passages • Reduces assay variability	• High costs

### Three-Dimensional Models and Coculture Systems

Two-dimensional (2D) monocultures are inherently unable to represent the complexity of *in vivo* cardiac structure, dynamics, and microenvironment. The development of 3D models and coculture systems could help to better mimic antifibrotic effects *in vitro*.

Multiple 3D cardiac organoid systems have been described (reviewed in Zuppinger, 2019). Organoids can be generated from induced pluripotent stem cells (iPSCs) that can be differentiated into different cardiac cell types, such as cardiomyocytes, endothelial cells, and fibroblasts (Filippo Buono et al., 2020). Alternatively, organoids can be generated from patient-derived primary cells, which of one or several cell types. Combinations of iPSCs, primary cells, and/or cell lines also exist. As 3D cardiac organoids can better recapitulate structure, cell composition, and function of heart tissue, they improve upon current multi-scaled drug screening platforms including *in vitro* assays and modeling diseases like cardiac fibrosis (Nugraha et al., 2018). The 3D models are currently being developed to fully recapitulate the composition of ECM, spatial distributions of cells, architectural organization of ECM, and cell crosstalk. Thus, 3D cardiac organoids are an attractive alternative *in vitro* model for cardiac diseases and HF. They are a promising tool for drug screening and assessing cardiotoxic effects, proliferation, and cell viability (Polonchuk et al., 2017) and can potentially contribute to the development of more physiologically relevant preclinical platforms to better predict *in vivo* drug efficacy. They can also be used for drug discovery for personalized medicine (Nugraha et al., 2019).

Coculture systems of different cardiac cell types have been developed both in 2D and 3D. Coculture of multiple cardiac cell types can better model disease features such as cardiac fibrosis (Baudino et al., 2008). However, finding physiologically relevant coculture models to develop cardiac fibrosis assays is challenging. Among others, it needs to identify optimal cell types (Pinto et al., 2016; Litvinukova et al., 2020), culture conditions (Zuppinger, 2019), and coculture cell ratios (Pinto et al., 2016), so that the coculture better mimics the *in vivo* situation.

From the perspective of an assay, it is important to understand whether a 3D and/or coculture model has greater physiological relevance, i.e., predictivity, for cardiac fibrosis. To adequately compare models, it is essential to use holistic, integrative readouts, i.e., a better model might show more *in vivo*-like (Kong et al., 2014) gene expression (Chothani et al., 2019), (Liu et al., 2017) protein expression, (Gabbiani et al., 1971) organoid structure and cellular composition (Lee et al., 2019), or (Skalli et al., 1989) more *in vivo*-like functional response, that is, higher predictivity for known modulators of cardiac fibrosis (Palano et al., 2020).

We are currently on the brink, where not only 2D but also 3D and coculture models are constantly improved and display their greater relevance. They will soon reach sufficient throughput, cost-effectiveness, and robustness for applications in large-scale drug screening.

## High-Content Imaging

### High-Content Imaging to Measure Cardiac Fibrosis *in vitro*

High-content imaging is a particularly attractive method to assay complex phenotypes such as cardiac fibrosis. Traditionally, HCI has been used as a cost-effective way to assess one or a few readouts that are thought to be most relevant for the process, e.g., antibody staining for α-SMA levels or certain forms of collagen as a proxy for cardiac fibrosis (see section “Readouts of Cardiac Fibrosis Assays”). Nowadays, staining, imaging, and image analysis can be automated in multi-well format, which allows to minimize the number of precious cells, reduces the number of reagents needed, and, thus, reduces costs, while at the same time increasing throughput and robustness. Modern image analysis methods allow us to integrate hundreds of parameters from microscopy images, i.e., creating a phenotypic profile instead of a single-marker readout [reviewed in Warchal et al. (2016); Scheeder et al. (2018)].

### A Phenotypic High-Throughput *in vitro* Cardiac Fibrosis Assay

We recently established cell culture conditions that promote deposition of mature collagen from primary human CFs *in vitro* (Palano et al., 2020). Based on these conditions, we set up a high-content immunofluorescence assay allowing for high-throughput, phenotypic identification of compounds with antifibrotic activity (Palano et al., 2020). Features extracted from the microscopy images, such as fluorescence intensity, cellular morphology, and staining texture, served as a basis for a linear classifier to classify cells into fibrotic or non-fibrotic phenotype. The assay can be run at low costs in 96-well or 384-well format, allowing to robustly assess the values of dose dependency and half-maximal effective concentration (EC50) potency for potential antifibrosis *in vitro*. Our *in vitro* assay correctly identified compounds with reported antifibrotic effects *in vivo*, targeting diverse cellular pathways (Palano et al., 2020), thus, highlighting its utility for high-throughput screening to discover novel compounds and targets for the treatment of cardiac fibrosis. The HCI assay has several advantages, i.e., (1) screening in high-throughput; (2) analyses in microwell format requiring fewer cells and minimizing the amounts of reagents; and (3) quantification of multiparametric readouts on a single-cell basis. In comparison with other traditional approaches, the *in vitro* cardiac fibrosis assay coupled with HCI analysis is ideal for large-scale screening and machine learning (ML)-based drug discovery. However, the *in vitro* phenotypic assay might not be able to fully reflect *in vivo* conditions of cardiac fibrosis. From the perspective of drug discovery, the hit compounds identified in our *in vitro* cardiac fibrosis assay need to be validated in more complex systems, such as 3D models, that could better mimic antifibrotic effects *in vivo*. In this way, the most promising candidates can be identified and selected for further characterization in animal models of cardiac fibrosis.

### Perspective and Outlook

The incorporation of multiple complex features instead of a single-marker readout reduces the intrinsic bias of any screening assay toward its readout (Abraham et al., 2014). These profiles can be analyzed by ML-based classification of the fibrotic phenotype on a well or single-cell level. HCI-based phenotypic classification also biases an assay toward the desired phenotype that the experimenter used to train the classification algorithm. While unsupervised ML approaches can partly mitigate this bias (Grys et al., 2017), robust validation of the control conditions (e.g., fibrotic and non-fibrotic) by orthogonal methods is essential to ensure that the phenotype, thus, hit compounds, and targets will be biologically relevant (Scannell and Bosley, 2016; Moffat et al., 2017).

Phenotypic drug discovery approaches are especially well suited to discover novel targets or novel compound mechanisms of action. Often, however, it is very resource intensive to deconvolute the hits from a screen. A combination of phenotypic and multiparametric assays, with clustered regularly interspaced short palindromic repeats (CRISPR)-based library screening, provides an attractive alternative. Turner et al. (2020) recently described an image-based CRISPR screening to identify regulators of kidney fibrosis. The authors used α-SMA as their main readout, combined with fibroblast proliferation and collagen expression, to identify genetic hits and novel targets in kidney fibrosis (Turner et al., 2020).

Major advantages of HCI compared with, for example, transcriptomics or proteomics are its lower cost and single-cell resolution. As such, HCI is well suited also for the analysis of 3D models and coculture systems. The increased complexity of these models requires single-cell resolution and analysis of diverse morphological characteristics to leverage their higher physiological relevance. In the long run, incorporation of multiplex staining mass cytometry could further increase the number of cellular markers and analyzed parameters while providing subcellular resolution, further reducing the number of precious cells needed (Giesen et al., 2014). However, 3D models still present major challenges for whole-mount imaging, data storage, image analysis, and data interpretation, warranting investment and further research studies to demonstrate their value to improve drug discovery (reviewed in Booij et al., 2019). The 2D and 3D models need to be carefully compared for their predictive value of *in vivo* efficacy.

Application of HCI and image analysis using ML-based classifiers, as in our phenotypic cardiac fibrosis assay (see section “A Phenotypic High-Throughput *in vitro* Cardiac Fibrosis Assay”; Palano et al., 2020), provides a way forward to develop such an assay toward a coculture and/or 3D model. Having set up a robust biochemical validation and a toolbox of known *in vivo* modulators of cardiac fibrosis allows for direct comparison and assay optimization toward increased predictivity of *in vivo* outcomes and, thus, increased physiological relevance.

It is attractive to envision a multiparametric assay, such as our HCI assay, as the basis of a screening cascade for novel treatments of cardiac fibrosis. The combination of HCI and ML provides a low-cost high-throughput assay for either screening chemical libraries for new equity or screening CRISPR libraries to identify new targets. Counterscreening for TGF-β pathway modulation could be a second level to provide an initial indication of the mechanism of action. Hits could then be profiled further downstream the cascade in coculture and/or 3D model that builds on similar HCI/ML principles before going *in vivo*.

Another interesting direction would be to develop multiparametric assays for different pathological models. Fibrosis is a hallmark of different pathologies of HF. Tool compounds that modulate specific phenotypes of fibrosis *in vivo* could be used to train ML models to recognize and predict specific mechanisms of actions in *in vitro* assays. Also, here continued assay development will lead the way, such as to choose physiologically relevant stimuli, cell composition, i.e., coculture, and/or pathology-specific patient cells, and to choose the best readouts to capture different pathologies of HF.

## Future Directions

### What We Still Need to Know About Fibroblasts

To better characterize *in vitro* models of cardiac fibrosis and to get more reliable and translational results, it is important to have a better understanding of the biology of CFs. Cardiac fibrosis is an extremely complex process, and, on one hand, blocking it to restore cardiac function and prevent disease progression into HF is the ultimate aim. On the other hand, once cardiac fibrosis is established, the question is to what extent it can be reversed. A recent study, examining the possibility of phenotypic reversion of fibrosis, found that by inhibiting TGF-β1 receptor kinase in CFs isolated from cardiac tissue collected from patients, they could promote dedifferentiation of myoFBs and reduce the expression profile of certain profibrotic genes (Nagaraju et al., 2019).

But the question remains whether these findings would translate to an *in vivo* scenario. Due to their phenotypic plasticity, fibroblasts can be reverted to a quiescent phenotype. Recent studies have shown that CFs can be maintained in a resting state *in vitro* using elastic silicone substrate (Landry et al., 2019) or can be reversed by culturing them on soft hydrogels or blocking them with inhibitors (Gilles et al., 2020). Using small molecule inhibitors, cardiac fibrosis can be attenuated in mouse models (Wang et al., 2018).

However, the challenges of reversing fibrosis go beyond the properties of fibroblast itself, but they extend to the reabsorption of the ECM by reducing expression of α-SMA and production of collagen (Shinde et al., 2017) and to the restoration of the cardiac structure. For this to work, cooperation between all cardiac cell types is needed.

### Current *in vitro* Assays Driving Drug Development

Currently available *in vitro* assays have contributed to drug candidates targeting cardiac fibrosis, such as TGF-β inhibitors, renin–angiotensin–aldosterone system (RAAS) inhibitors, endothelin inhibitors, MMP inhibitors, relaxin, and others (reviewed in Fang et al., 2017). Their failure in clinical trials highlights the need for more physiologically relevant assays to discover new antifibrotic drugs.

In a recent study, a novel antifibrotic therapeutic based on a naturally derived substance was developed using two different hypertension-dependent rodent models (Schimmel et al., 2020). These antifibrotic drug candidates were identified by functional screening of 480 chemically diverse natural compounds in primary human CFs, subsequent validation, and mechanistic *in vitro* and *in vivo* studies. High-throughput natural compound library screening identified 15 substances with antiproliferative effects. Hits were analyzed for dose-dependent inhibition of proliferation, modulation of apoptosis, and expression of ECM. Using multiple *in vitro* fibrosis assays and stringent selection algorithms, the authors identified the steroid bufalin and the alkaloid lycorine to be effective antifibrotic molecules both *in vitro* and *in vivo*. These *in vitro* findings were confirmed *in vivo* with an angiotensin II-mediated murine model of cardiac fibrosis in both preventive and therapeutic settings and in the Dahl salt-sensitive rat model (Schimmel et al., 2020). Another study, using NIH 3T3 mouse fibroblasts, demonstrated that long noncoding RNA (lncRNA) Neat1 has important functions for fibroblast survival, migration, and proliferation. These findings were confirmed in a MI model using the genetic loss of Neat1 mice (Kenneweg et al., 2019). Likewise, noncoding RNAs, such as miRNA-21, miR-208a, and Meg 3, have recently emerged as promising targets (Piccoli et al., 2017; Kenneweg et al., 2019). Studies like these promote new angles for therapeutic approaches with regard to antifibrotic agents and highlight the translational importance of well-established *in vitro* assays for the discovery of novel targets and development of drugs against cardiac fibrosis.

## Conclusions: Where Are We Heading?

The switch from immortalized cell lines to primary cells, cocultures, and 3D models is a step forward in filling the translational gap. The use of more physiologically relevant *in vitro* models in preclinical development will lead to drugs having better efficacy and safety parameters *in vivo* and in the clinic. For *in vitro* effects to translate to *in vivo*, it is essential that the *in vitro* assay readout is predictive for *in vivo* effects. For cardiac fibrosis, assays with single, well-defined readouts have shown only limited translatability toward *in vivo*. Multiparametric readouts, such as omics or high-content techniques, provide a way forward, integrating a holistic, phenotypic fibrotic response. HCI provides a multiparametric readout at low costs, suitable for high-throughput screening, and it allows analysis on a single-cell level thus supporting analysis of cocultures and 3D models. These advantages make HCI and ML-based data analysis our method of choice for multiparametric assessment of cardiac fibrosis. As these new approaches come not without challenges and the need for investments, there remains a strong need for these technologies and each *in vitro* assay setup to clearly demonstrate improved predictivity toward *in vivo* and ultimately translation to the clinics. Ideally, *in vitro* assay setups are cross validated with collections of molecules that have confirmed the benefits of treating cardiac fibrosis *in vivo*. In addition, Booij et al. (2019) suggested that multiparametric phenotypic approaches allow us to determine a footprint of successful medicines and optimize new drugs toward this.

Multiple different pieces are coming together toward developing a physiologically relevant, predictive, and high-throughput *in vitro* assay for novel treatments against cardiac fibrosis. While the ideal will likely never be reached, the substantial improvements made in recent years will lead to a better and earlier prediction of drug efficacy and identification of novel antifibrotic drug mechanisms and, thereby, will reduce failures, cost, and time in clinical trials.

## Author Contributions

All authors were involved in the design and writing of this manuscript.

## Conflict of Interest

AF and EM are employees of AstraZeneca. The remaining author declares that the research was conducted in the absence of any commercial or financial relationships that could be construed as a potential conflict of interest.

## Abbreviations

3D: three-dimensional
AI: artificial intelligence
ECM: extracellular matrix
TGF- β: transforming growth factor-beta
PDGF: platelet-derived growth factor
TNF α: tumor necrosis factor-alpha
HF: heart failure
IL-1 β: interleukin-1 beta
TAK1: transforming growth factor- β-activated kinase 1
JNK: c-JunN-terminal kinase
PI3K: phosphoinositide 3-kinase
RhoA: Ras homolog family member A
α-SMA: alpha-smooth muscle actin
MMPs: matrix metalloproteinases
CCN2: connective tissue growth factor
RT-qPCR: real-time quantitative polymerase chain reaction
IF: immunofluorescence
ELISA: enzyme-linked immunosorbent assay
WB: Western blot
PICP: procollagen type I carboxy-terminal propeptide
SEM: scanning electron microscopy
HPCL: high-performance liquid chromatography
LC-MS: liquid chromatography-mass spectrometry
MS: mass spectrometry
iPSCs: induced pluripotent stem cells
HCI: high-content imaging
RAAS: renin–angiotensin–aldosterone system
ML: machine learning.
